# The Relation of Adenoids and Tonsils to Mental Deficiency

**Published:** 1915-02

**Authors:** 


**The Relation of Adenoids and Tonsils to Mental Deficiency.** Arthur M. Corwin, Chicago, "Am. Jour. Oph. & Oto-Laryn.," Dec., 1914. Beside the morbid adenoid there is no other affection of childhood that is so common and far reaching in its malign influence upon comeliness, comfort, reliability, and individual usefulness. It is the direct and remote results of the abnormal presence of lymphatic tissue in the pharyngeal vault as well as between the faucial pillars upon mental and moral integrity that are especially damning. Among four hundred children Bonazzolo found that one hundred and forty-one displayed great lack of the power to concentrate attention and therefore were backward students. In all but twenty-four of these adenoids existed, and these had nasal obstruction from other causes. In New York it was found that in a school population of 650,000 thirty per cent. of the children were about two years behind their grades, and that ninety per cent. of these were delinquent because of removable eye, ear, nose, or throat lesions. One hundred and fifty marked defectives were very backward in their studies and incorrigible in character. 137 of them had enlarged tonsils and adenoids. These were removed and in six months they were re-examined. All were doing well in studies and their characters had undergone marked improvement. It has been estimated that forty thousand children annually in Minnesota have adenoids that retard them one year in work. It costs this state one million dollars extra per annum to educate these retarded children, an amount easily saved by a simple operation. The lack of attention and lowered power of memory resulting from typic adenoid obstruction and irritation are responsible for an enormous amount of mental backwardness. The child falls behind his fellows and fails to catch up; he is accounted a dunce; he is punished by his teachers and his parents, ridiculed by his mates, and soon finds other matters to take his attention. He grows sullen, apathetic, or mischievous, and is considered incorrigible and a nuisance. "Straight road from adenoids to penitentiary."

In eighty five to ninety per cent. of all adenoids there is involvement of the eustachian tubes, with deafness of varying degree. The vast majority of these unfortunates are stunted in mind, not because of initial organic deficiency in brain cells, but because of faulty nutrition and want of proper training. Blake of Boston found decided deafness in 39 out of 47 children examined and after removal of adenoids 35 showed marked improvement in hearing.

The removal of adenoids is not put forward as a panacea for all juvenile delinquency and retarded development, but our plea would emphasize the fact that a multitude of children with normal brains and normal means of developing them, if denied proper surgical attention, will suffer deterioration of their normal gray matter. They may not graduate into criminals or degenerates or public dependents, yet they will be seriously handicapped all their lives.—H. W. C.

